# Types and Sources of Social Support Accessible to University Students with Disabilities in Saudi Arabia during the COVID-19 Pandemic

**DOI:** 10.3390/healthcare11040464

**Published:** 2023-02-06

**Authors:** Ahmed A. Ahmed, Ali A. Almishaal, Sehar-un-Nisa Hassan, Reham M. Kamel, Mohammed Raafat M. Atteya, Sofian T. Obeidat, Hesham S. Abdelmaguid, Abdullah A. Alanazi, Liza Mendizabal Villacorte, Fahad D. Alghatani

**Affiliations:** 1 Department of Social Sciences, College of Arts, University of Ha’il, Ha’il 81451, Saudi Arabia; 2 Department of Working with Individuals and Families, Faculty of Social Work, Helwan University, Helwan 11795, Egypt; 3 Department of Speech-Language Pathology and Audiology, College of Applied Medical Sciences, University of Ha’il, Ha’il 81451, Saudi Arabia; 4 Department of Public Health, College of Public Health and Health Informatics, University of Ha’il, Ha’il 81451, Saudi Arabia; 5 Department of Psychiatry, Cairo University, Cairo 11651, Egypt; 6 Department of Physical Therapy, College of Applied Medical Sciences, University of Ha’il, Ha’il 81451, Saudi Arabia; 7 Department of Basic Sciences, College of Preparatory Year, University of Ha’il, Ha’il 81451, Saudi Arabia; 8 College of Nursing, University of Ha’il, Ha’il 81451, Saudi Arabia

**Keywords:** disability, emotional support, sources of social support, university students, COVID-19 pandemic

## Abstract

University students with disabilities face an increased risk of experiencing negative implications in educational, psychological, and social spheres during the COVID-19 pandemic. This study aimed at assessing various dimensions of social support and its sources during the COVID-19 pandemic that availed university students with disabilities. This cross-sectional descriptive study collected data from 53 university students with disabilities. We administered the Social Support Scale (SSC) to assess five dimensions: informational, emotional, esteem, social integration and tangible support, and access to social support from four sources: family, friends, teachers, and colleagues. Multiple regression analysis showed that university students with disabilities mainly relied upon their friends for informational support (β = 0.64; *p* < 0.001), emotional support (β = 0.52; *p* < 0.001), and social integration support (β = 0.57; *p* < 0.001). Family members (β = 0.406; *p* < 0.01) and colleagues (β = 0.36; *p* < 0.01) provided esteem support to students with disabilities. Support from teachers demonstrated an association with informational support (β = 0.24; *p* < 0.05). The findings from the current study suggest that students with disabilities primarily sought informational, emotional, and social integration support from their peers. Although teachers were the primary source of informational support, emotional and esteem support were not found to be significantly associated with them. These findings necessitate exploring the underlying factors and how to enhance them during unusual circumstances such as online distance education and social distancing.

## 1. Introduction

International organizations such as the Disability Council International (DCI), International Disability Alliance (IDA), and local agencies in various countries, including Saudi Arabia, are striving to address the issues faced by individuals with disabilities. These organizations actively seek to provide the necessary care services, educate families in addressing the needs of people with disabilities adequately, and provide the necessary support to cope with psychological pressure. Furthermore, human rights activists propagate that providing care to people with disabilities is no longer a choice but a legitimate right.

The COVID-19 pandemic presented a unique challenge to individuals with disabilities because the environmental quality of life was compromised during the pandemic, thereby increasing their vulnerability to experiencing a poor physical, mental, emotional, social, and psychological quality of life [1,2]. The risk of social isolation and dependency multiplied during the pandemic for this vulnerable population, especially those who faced poor adaptation to their social and family environment. Consequently, they encountered health, psychological, social, and educational problems [3,4]. It is, therefore, essential to assess and address the problems faced by individuals with disabilities and enable them to live positively and productively along with other community members despite their disability condition.

The current study was conducted in Ha’il, a city in the northern region of Saudi Arabia. The population of Ha’il city is approximately 267,005 and according to the Disability Population Survey (2017), the rate of disability in the Ha’il region is around 3% [5,6]. The Saudi government respects the right to education for individuals with disabilities as guaranteed by international conventions and treaties to human beings. The government of Saudi Arabia has developed several criteria and conditions to enhance access to higher education for individuals with disabilities. The Supreme Council for the Affairs of Persons with Disabilities in Saudi Arabia asserts the provision of medical, preventive and habilitative services, education, recruitment and employment, easy integration into various facets of public life, cultural activities, deployment of sports facilities, and access to information technology. Around 17% of people with disabilities study at the university level in Saudi Arabia [6,7].

University students with disabilities are an integral part of the higher education system. Therefore, universities strive to support students with disabilities due to their vulnerability. The type of assistance provided to students with disabilities is related to meeting the study requirements from admission to university until graduation. The services provided to university students with disabilities have a significant role in alleviating disability, and these services must be integrative. Educational institutions should consider the person with a disability as an individual living in society rather than a person living in a vacuum, as recommended by the previous literature [8,9]. 

University education must prepare students with disabilities to deal with the challenges in their personal lives and equip them with the necessary skills to contribute to the labour market and be employable for jobs commensurate with their needs. The goal is to reduce the burden on society and the health system, thus limiting the physical, social, environmental, and psychological repercussions of their disability on their lives. 

Social support plays a vital role in empowering individuals and promoting equality in society in general [10]. The availability of social support for individuals with disabilities is even more critical because it helps them cope with the pressures associated with disability [11]. The social support system may reach through immediate and extended family members, friends, fellow students, coworkers, and the community at large [10]. Perceived social support is a belief that the surrounding environment is a source of support. Social work has broadly adopted the concept of social support to describe some of the activities used by social workers with their clients to strengthen some aspects of their lives and as a defensive activity for vulnerable groups. This support takes one of the following images: the possibility of having assistance when needed, compensation in the sense of providing some services as an alternative to traditional services that individuals with disabilities lack, or change in the sense of allowing individuals with disabilities to change themselves to conform to life conditions [12]. 

Currently, there is limited empirical evidence from the Middle East region about the types of social support available to university students with disabilities during the COVID-19 pandemic. During the COVID-19 pandemic, most of the studies that assessed social support and psychological health focused on all students in higher education rather than on students with disabilities [13,14]. These studies employed a cross-sectional design and collected data by using electronic means and thus did not gather specific experiences of students with disabilities. 

One study from the pre-COVID-19 pandemic period compared the learning experiences and levels of social satisfaction among students with disabilities from four countries including Yemen, Oman, Jordan, and Qatar [15]. The findings revealed that there is a wide gap in terms of quality and services provided to university students with disabilities in the universities. Overall, students experience higher levels of social satisfaction compared with psychological, educational, and environmental satisfaction. Previous studies, most of which focus on individuals with disabilities, reported that individuals with disabilities experienced low levels of perceived social support in all dimensions including functional, instrumental, emotional, and structural [16]. Research from Nigeria suggests that people with disabilities in this country face many challenges due to negative perceptions associated with disability and there is a need for functional social support. This form of social support focuses on altering the perception and attitudes of the person living with a disability [17]. 

Research from other parts of the world has shown that parents and friends are significant sources of social support for university students with disabilities. Moreover, the literature reports that students with mental disabilities had considerably fewer sources of social support than others [18]. One study based on college students with disabilities demonstrated that support from parents and peers is associated with academic success and social support satisfaction [19]. A scoping review of studies showed that formal social support is available primarily from educational institutions to children and young adults with disabilities in low- and middle-income countries. These include language and speech therapy services, school psychological services, school counselling services, school health services, school social work, and in some cases, vocational skills services [20]. It is crucial to increase the prospects of university education for students with disabilities along with appropriate social support, psychological counselling, and rehabilitation services, and it is considered a humanitarian and social duty. Recent studies have underlined the need to provide psychological and informational support to students with disabilities studying in universities during the COVID-19 pandemic [21,22].

Assessment of access to social support for students with disabilities during the COVID-19 pandemic has become an important area of research due to its unique context, which necessitates a shift to complete online education for a relatively longer duration. Previous research has shown that among social factors, access to a wide range of social support sources and the types of social support accessible could influence students’ retention in higher education and academic accomplishment [23]. In a nutshell, social support is experiencing acceptance and receiving care, assistance, or help from social support networks when needed. Researchers have identified various components of social support that include an attachment to social relations, integration with the social network, the opportunity for growth, receiving appraisal, guidance, and a sense of being a contributor or needed by others [24]. In addition, access to perceived and received social support is negatively associated with stress in university students as reported in the literature. It thus plays a significant role in promoting health and well-being [25]. 

Therefore, the current study aims at understanding the gaps in providing social support to students with disabilities studying in a higher educational institute in Saudi Arabia during the period of complete lockdown and online studies in the Ha’il region. 

This study aimed at exploring the types of social support accessible to university students with disabilities during the COVID-19 pandemic in Saudi Arabia. Moreover, it assessed which sources of social support (family, friends, teachers, and colleagues) significantly contributed to the provision of various types of support, including informational, emotional, esteem, social integration, and tangible support. We hypothesized that (1) friends and family would be the primary source of emotional and esteem support; (2) teachers and colleagues would be the primary sources of informational and tangible support; and (3) friends and colleagues would be the main source of social integration support. 

## 2. Materials and Methods

### 2.1. Study Design

The study adopted a cross-sectional research design. The target population was university students with a disability who studied in undergraduate programs during the COVID-19 pandemic in Saudi Arabia. Participants were selected through a purposive sampling method, and the study sample comprised 53 students with disabilities. The participants were recruited from higher education institutions in the Ha’il region of Saudi Arabia. 

### 2.2. Procedure

Students were invited to participate in the study through their university email addresses. No incentives were offered to students, participation was voluntary, all students completed the informed consent form before the interview, and those who consented to participate were interviewed in one-to-one settings. Responses were recorded on a printed copy of the study questionnaire. The study questionnaires did not include any questions that may reveal the identity of students. The study was completed in approximately six months between June 2020 and December 2021. 

### 2.3. Study Measures

The study questionnaire consisted of two sections. The first section comprised a set of items to collect data on demographic and background variables including gender, age, field of study, year of study, and type of disability. The second part of the questionnaire included social support scale to measure various dimensions of social support and sources of social support. 

### 2.4. Types of Social Support and Sources of Social Support

The measure Social Support Scale (SSC) was used to assess types of social support and sources of social support. Items on this measure were adapted from previous measures of social support [26,27]. These items tap various dimensions of social support in the psychological literature [28,29]. 

Psychometric analysis of data demonstrated that this measure assesses the five dimensions of social support, namely, (a) informational support, (b) emotional support, (c) esteem support, (d) social integration, and (e) tangible support. Informational support refers to sharing of information and advice to help individuals understand and solve the problems at hand. Emotional support refers to providing support by listening and understanding the feelings of other individuals. Esteem support involves expressing encouragement and trust in the abilities of other individuals. Social integration support refers to the availability of support to develop connections and opportunities for interaction with individuals who share similar interests and activities. Finally, tangible support refers to practical support, such as sharing work or responsibilities to help individuals manage the issues. Each subscale comprises five items, and each item is rated on a three-point scale from (3 = Agree; 2 = Neutral, and 1 = Disagree). The minimum score on each subscale is 5 and the maximum score is 25. A higher score on the sub-scale indicates high access to that type of support. 

The sources of social support were categorized into four areas, including support from family, support from friends, support from teachers, and support from colleagues. A set of five items measures each sub-category of the sources of social support, and a higher score on the scale indicates more accessibility to social support from that source. 

The sample items and alpha reliabilities on all subscales of sources of social support are also reported (Supplementary File A). The values of Cronbach’s alpha on all sub-scales of social support and sources of social support lie above 0.70 and below 0.80.

### 2.5. Data Analysis

We performed the statistical analysis using SPSS 25.0 version for Windows (SPSS Inc., Chicago, IL, USA). The data on background variables is categorical and descriptive analysis is reported as frequency and percentage values. The data on types of social support and sources of social support is continuous and descriptive data is reported with mean scores along with 95% CI. The data on study variables were checked for normality assumptions using Kolmogorov–Smirnov and Shapiro–Wilk tests (Supplementary File A). Moreover, the values of skewness and kurtosis were computed. The findings showed that data on some variables (informational support, emotional support and support from friends, teachers, and colleagues) meet the normality assumptions. The data on four variables (self-esteem support, social integration support, tangible support, and support from family) did not meet normality assumptions. A two-step transformation to normality was completed by retaining the original mean score values and standard deviation values that allow the accurate interpretation of results (Supplementary File A). Pearson correlations were applied to determine the relationship between sources of social support and types of social support. The data met the assumptions of multiple linear regression analysis that includes independent observation, normality, linear relationship, and absence of multicollinearity. The values of VIF were below 4 and tolerance values lie above 0.25, indicating the absence of multicollinearity. For multiple regression analysis, the statistical significance was chosen at a *p*-value less than or equal to 0.05. 

### 2.6. Ethical Approval

The study protocol was reviewed and approved by the Ethical Review Board of the University of Ha’il (Number: 25518-5-42). The study was completed following the Declaration of Helsinki for human subject research. All participants completed informed consent, and data anonymity was maintained to maintain the confidentiality of participants..

## 3. Results

The distribution of characteristics on demographic variables is presented in Table 1. The sample comprised 53 students with disabilities, among which 13 were male students (25%) and 40 were female students (75%). Approximately half of the students fall in the age group 20–22 years (n = 28; 53%), and 25 (47%) students are in the age category 23–26 years. Most students who participated in this research studied Arts and Humanities (n = 44; 83%). Around half of the students in this sample reported multiple disabilities (n = 25; 47%), followed by those with motor disabilities (n = 13; 25%), visual disability (n = 13; 25%), and hearing loss (n = 2; 3%).

Table 2 illustrates that the highest mean score was on esteem support (M = 10.91; S.D. = 2.48; 95% CI = 10.23–11.60), followed by social integration support (M = 10.72; S.D. = 2.82; 95% CI = 9.94–11.49). The informational support was the lowest (M = 10.26; S.D. = 2.61; 95% CI; 9.54–10.99) compared with other types of social support. The mean score on support from family was the highest (M = 10.83; S.D. = 2.58; 95% CI = 10.11–11.54), followed by support from friends (M = 10.45; S.D. = 2.39; 95%CI = 9.79–11.11), support from teachers (M = 10.26; S.D. = 2.16; 95% CI= 9.67–10.86), and then support from colleagues (M = 10.13; S.D. = 1.88; 95% CI = 9.12–10.65).

Table 3 shows that support from family was significantly associated with esteem support (r = 0.435; *p* < 0.01) and social integration support (r = 0.522; *p* < 0.01). Support from friends was significantly associated with informational support (r = 0.632; *p* < 0.01), emotional support (r = 0.520; *p* < 0.01), and social integration support (r = 0.647; *p* < 0.01). Support from teachers was significantly negatively associated with social integration support (r = −0.299; *p* < 0.05). Support from colleagues was found to be significantly associated with informational support (r = 0.356; *p* < 0.01) and esteem support (r = 0.465; *p* < 0.01).

Table 4 shows results from multiple linear regression analysis models where family, friends, teachers, and colleagues’ support was assessed as predictors of informational and emotional support for students with disabilities during the COVID-19 pandemic. Analysis revealed that the model’s predictor variables explain 51% variance for informational support. Moreover, support from friends (β = 0.64; *p* < 0.001) was the strongest predictor for informational support, followed by support from colleagues (β = 0.29; *p* < 0.01) and support from teachers (β = 0.24; *p* < 0.05). The overall model fit for emotional support was significant at *p* < 0.01, and a 21% variance in emotional support is explained by the predictor; social support from friends (β = 0.52; *p* < 0.001) was the only significant predictor for emotional support. 

Multiple linear regression analysis models were tested to determine the relationship between support from family, friends, teachers, and colleagues and esteem support and social integration support accessible to students with disabilities during the COVID-19 pandemic (Table 5). Analysis revealed that predictor variables explain the model’s 27% variance for esteem support. Support from family (β = 0.406; *p* < 0.01) and support from colleagues (β = 0.36; *p* < 0.01) were significant predictors for esteem support. The overall model fit for social integration support was significant at *p* < 0.001. A 59% variance in social integration support is explained by support from family (β = 0.37; *p* < 0.01), support from friends (β = 0.57; *p* < 0.001), and support from colleagues (β = −0.33; *p* < 0.01), which were found to be significant predictors for social integration support. 

## 4. Discussion

Students with disabilities encounter a wider range of stressors than students without disabilities due to their particular needs for communication and mobility. Thus, they are more likely to need help with access to information, completion of academic tasks, and networking with other students in university settings. These stressors may influence the capability of students with disability to continue their studies or academic performance. The literature demonstrated that different population segments were at increased risk of developing neurological symptoms [30], mental health problems, and psychiatric symptoms [31]. Therefore, access to social support from various sources, including family, friends, teachers, colleagues, and the wider community, helps buffer the adverse effects of stress and increases the probability of retention in their study programs [16,23]. Access to social support in various domains also determines this support’s broader and positive impact, especially in difficult times such as the COVID-19 pandemic. Therefore, the current study examined how various sources of social support relate to social support in various domains available to university students with disabilities during the COVID-19 pandemic. The current study was, therefore, designed to examine specifically how various sources of social support relate to social support in various domains that were available to university students with disabilities during the COVID-19 pandemic. Online studies were implemented in all universities in Saudi Arabia to prevent the spread of COVID-19 infection. In this study, most participants were female students within the age range of 20 to 26 years, and most were studying in arts, social sciences, and humanities disciplines. About half of the students in this sample reported having multiple types of disabilities, and the remaining reported hearing, motor, or visual disabilities. Adequate social support must be accessible to university students with disabilities because it may significantly contribute to academics [32]. It has been observed that individuals, despite their disabilities, with university degrees have better prospects of employment and are able to live healthier and more productive lives than those without higher education [33]. 

The descriptive findings of the current study demonstrated that among types of social support, the highest score was for esteem support. Esteem support can significantly contribute to retaining students’ courage and trust in their abilities and ultimately enables them to experience and feel pride in academic accomplishments. Another study has shown that encouragement and appreciation from teachers and including students with disability in intellectual discussions enhances positive educational outcomes for these students [34]. Our findings demonstrated that the lowest score was for information support. This finding raises concerns about the limited access to informational support during the COVID-19 pandemic. Due to the implementation of online studies, there is a high probability that students with disabilities need access to valid information to better understand and solve the problems at hand. Limited support may negatively affect academic experiences and the mental well-being of at-risk students with disabilities. A scoping review of the literature demonstrated that people with disabilities faced disparities in access to health services, education, and community services during the COVID-19 pandemic [35]. Poor informational support could be the consequence of such disparities and thus increases the vulnerability of at-risk populations to experiencing the psychosocial aftereffects of such emergencies. 

The current study’s findings also showed that among the sources of social support, the highest score was for support from family. This finding is explainable as collectivist culture and religious values generally prevail in Saudi society, and providing support to individuals with disabilities is considered an integral family role. Furthermore, this might have positive implications on student retention in higher education as the previous literature also revealed that family social support significantly influences students’ academic persistence in early education, college, and university [19]. 

The previous literature has also shown that students likely feel marginalized when they see themselves as not fitting into the university community, which has repercussions for their emotional health and leads to attrition [36]. Our study findings demonstrated that support from friends is significantly associated with social integration support. Social integration results in positive impacts such as retaining an interest in studies and academic success. Support from friends who share similar interests and activities during the period of the COVID-19 pandemic was not only beneficial in terms of academic outcomes. The current study also showed that friends were a significant source of emotional support for students with disability. These findings align with previous research showing that friends comprise an essential source of social support for students in community colleges [35,36,37]. The COVID-19 pandemic increased the risk of experiencing depression, anxiety, stress symptoms, and poor quality of life. 

In contrast, access to social resources negatively associates with feelings of distress in the general population [38]. Thus, for students with disabilities, access to emotional support from their friends could be a practical resource to mitigate the impacts of social isolation. Furthermore, both emotional support and social integration from the network of friends in university settings are valuable social resources for building healthy personal relationships, decreasing the risk of experiencing academic stress, and enhancing the chances of achieving academic goals. 

In multivariate analysis of our data, we found that support from teachers was not significantly related to emotional support, esteem support, or social integration support. The possible explanation for this non-significant association might be the context of the COVID-19 pandemic, when social distancing measures and distance education were implemented. Other researchers have also identified that students with disabilities faced structural barriers to learning during the COVID-19 pandemic [39], students living in remote areas found it especially difficult to adapt to online education due to poor internet access, non-availability of related services, and poor social support, resulting in an increased risk for mental health difficulties among vulnerable populations. Teachers had limited direct interaction with students in the physical environment and uncertainty was pervasive. These factors might have restricted the opportunities to show empathy, concern, and supportive care. These findings significantly contribute to expanding the existing knowledge base and provide useful information and insights for educational support interventions that need to be designed by university personnel for online studies. Previous research also suggests that students with disabilities in educational institutions in Saudi Arabia often graduate at a much lower rate. People with disabilities were at an increased risk of social isolation [40]. The literature demonstrated that the mental health care system faced many challenges in providing adequate care to vulnerable populations [41]. There is a strong need for a network of academic and social support measures necessary to cater to the diverse needs of students in higher education and enable students with disabilities to experience academic success and psychological health. 

The Saudi government, following its Vision 2030, is committed to providing adequate educational support services to students with disabilities to promote self-reliance and enhance their active participation in community development and progress [42]. There are currently several gaps in research in this area from Saudi Arabia. For instance, there is a need to acquire data to identify factors such as health behaviours and lifestyles of people with disabilities that may influence academic persistence. Furthermore, efforts are needed to explore current gaps in the use of online education systems for students with disabilities and what assistive technologies are needed to enhance engagement and learning outcomes for students with disabilities at varying educational levels. 

Study Limitations and Future Research Directions

Some of the limitations of the current study can also be addressed in future research, such as the limited sample size and the data collected from educational institutes in the Ha’il region. Moreover, the sample was not probabilistic but convenient. This sample size should be expanded to other regions because education and technology reforms are taking place quickly in Saudi Arabia. Therefore, the experiences of students seeking higher education in more developed regions of Saudi Arabia, such as Riyadh and Jeddah, are likely different. The study did not include students with learning disabilities. Moreover, the current study only focused on accessibility to social support and did not study several other psychological and environmental factors that may have influenced the student’s capability to cope with the demands of higher education during the pandemic. The qualitative data for these variables could give further clarity directly from the university students’ perspectives. Therefore, it is recommended that future research should focus on qualitative inquiry to collect data about coping levels and the nature of the difficulties experienced by university students with disabilities during the COVID-19 pandemic and online studies. How do these difficulties impact on the quality of social interaction with fellow students, teachers, and other community members? There is also a need to collect data from teachers’ perspectives such as the barriers faced by them in providing adequate tutoring to students with disabilities during online education.

## 5. Conclusions and Recommendations

The findings from the current study suggest that university students with disabilities primarily relied upon their friends to seek emotional support and social integration support. Family members and colleagues mainly provided esteem support. Both friends and teachers were significant sources of informational support. Online education and social distancing measures during the COVID-19 pandemic in Saudi Arabia might have restricted teachers from sufficiently meeting the emotional and esteem support needs of university students with special needs, which necessitates exploring how it can be enhanced during unusual circumstances. Currently, there are several gaps in existing research on the educational experiences of students with disabilities during the COVID-19 pandemic that warrant further investigation. There is a wide range of social and psychological factors on both students’ and teachers’ sides that may have influenced academic processes and outcomes during this crisis and their repercussions on students’ health, well-being, academic retention, and achievement. 

It is recommended that initiatives should be taken at institutional and country levels to develop an action plan to enhance access to informational, emotional, esteem, and social integration support for university students with disabilities during unusual circumstances such as pandemics. This action plan should focus on the capacity building of teachers for the implementation of social support interventions for students with disabilities. Even though there is generally a positive attitude from academic staff towards university students with disabilities, there is a need to increase awareness among teachers about the provision of adequate emotional support for students with disabilities during any circumstances when direct physical interaction is not possible such as a lockdown or online studies. Education information management systems can be employed to identify at-risk students according to the type or severity of disability and should be supported through tailored programs.

## Figures and Tables

**Table 1:** Demographic characteristics of university students with disabilities (n = 53).

Variables	Categories	*n*	(%)
Gender	Male	13	25%
	Female	40	75%
Age	20–22 years	28	53%
	23–26 years	25	47%
Field of Study	Health Sciences	2	4%
	Engineering	7	13%
	Art and Humanities	44	83%
Types of Disability	Motor	13	25%
	Hearing Loss	2	3%
	Visual Disability	13	25%
	Multiple Disabilities	25	47%
	First Year	9	17%
Year of Study	Second Year	21	39%
	Third Year	13	24%
	Fourth Year	10	19%
Type of rehab services used by students from the university	Physical Health	16	30%
	Social Rehabilitation	11	21%
	Psychological Rehabilitation	26	49%

**Table 2:** Mean scores on types and sources of social support accessible to university students with disabilities during the COVID-19 pandemic in Saudi Arabia (n = 53). Values of skewness and kurtosis of all study variables are reported in Table S3 in Supplementary File A.

Types of Social Support *	Mean (S.D.)	95% CI
Information Support	10.26 (2.61)	9.54–10.99
Emotional Support	10.43 (2.22)	9.82–11.05
Esteem Support	10.91 (2.48)	10.23–11.60
Social Integration Support	10.72 (2.82)	9.94–11.49
Tangible Support	10.69 (1.94)	10.35–11.42
Sources of Social Support *	**Mean (S.D.)**	**95% CI**
Support from Family	10.83 (2.58)	10.11–11.54
Support from Friends	10.45 (2.39)	9.79–11.11
Support from Teachers	10.26 (2.16)	9.67–10.86
Support from Colleagues	10.13 (1.88)	9.12–10.65

* Each sub-scale comprises five items.

**Table 3:** Pearson correlation between sources of social support and types of social support accessible to students with disabilities during the COVID-19 pandemic (n = 53).

	Types of Social Support				
Sources of Social Support	Information Support	Emotional Support	Esteem Support	Social Integration Support	Tangible Support
Support from Family	0.103	0.160	0.435 **	0.522 **	0.148
Support from Friends	0.632 **	0.520 **	0.035	0.647 **	0.099
Support from Teachers	0.242	0.040	−0.091	−0.299 *	0.028
Support from Colleagues	0.356 **	0.142	0.465 **	0.035	0.152

*p*-value significance: * *p* < *0*.0.5; ** *p* < 0.01;

**Table 4:** Results of multiple linear regression analysis to determine the relationship of support from family, friends, teachers, and colleagues with informational support and emotional support.

Informational Support				
Predictor Variables	B	Beta	*p*-Value	95% CI
Support from Family	−0.167	−0.165	0.177	−0.48
Support from Friends	0.702	0.641	0	0.46–0.93
Support from Teachers	0.3	0.249	0.022	0.04–0.55
Support from Colleagues	0.41	0.295	0.008	0.11–0.71
R-squared	0.553	F (4, 52)		
Adjusted R-squared	0.516	*p*-value (F)	0	
**Emotional Support**				
**Predictor Variables**	**B**	**Beta**	***p*** **-value**	**95% CI**
Support from Family	−0.03	−0.035	0.822	−0.53
Support from Friends	0.491	0.528	0.00	0.240–0.743
Support from Teachers	0.064	0.062	0.644	−0.552
Support from Colleagues	0.043	0.037	0.786	−0.639
R-squared	0.276	F (4, 52)		
Adjusted R-squared	0.216	*p*-value (F)	0.003	

B = unstandardized coefficient; *p*-value significance = *p* < 0.05.

**Table 5:** Results of multiple linear regression analysis to determine the relationship of support from family, friends, teachers, and colleagues with esteem support and social integration support.

Esteem Support				
Predictor Variables	B	Beta	*p*-value	95% CI
Support from Family	0.39	0.406	0.008	0.105–0.675
Support from Friends	−0.211	−0.203	0.123	−0.541
Support from Teachers	0.106	0.093	0.476	−0.593
Support from Colleagues	0.479	0.363	0.007	0.136–0.822
R-squared	0.33	F (4, 52)		
Adjusted R-squared	0.274	*p*-value (F)	0.001	
**Social Integration Support**				
**Predictor Variables**	**B**	**Beta**	***p*** **-value**	**95% CI**
Support from Family	0.405	0.371	0.002	0.163–0.647
Support from Friends	0.677	0.574	0	0.447–0.908
Support from Teachers	−0.205	−0.157	0.11	−0.865
Support from Colleagues	0.502	0.335	0.001	−1.005
R-squared	0.624	F (4, 52)		
Adjusted R-squared	0.593	*p*-value (F)	0.00	

B = unstandardized coefficient; *p*-value significance = *p* < 0.05.

## Data Availability

The data that support the findings of this study are available on request from the corresponding authors [S.-u.-N.H and A.A.A]. The data are not publicly available to maintain the confidentiality and privacy of research participants.

## Supplementary Materials

The following supporting information can be downloaded at: https://www.mdpi.com/article/10.3390/healthcare11040464/s1, The supporting information is available in Supplementary File A.

## References

[1] Scully J.L. (2020). Disability, Disablism, and COVID-19 Pandemic Triage. J. Bioethical Inq..

[2] Zahra A., Hassan M.S., Park J.-H., Hassan S.-U., Parveen N. (2022). Role of Environmental Quality of Life in Physical Activity Status of Individuals with and without Physical Disabilities in Saudi Arabia. Int. J. Environ. Res. Public Health.

[3] Al Awaji N., Aldhahi M., Akil S., Awad S., Mortada E. (2021). Quality of Life, Needs and Fears of Mothers of Children with Disabilities in Saudi Arabia during the COVID-19 Lockdown. Int. J. Environ. Res. Public Health.

[4] Al-Aoufi H., Al-Zyoud N., Shahminan N. (2012). Islam and the cultural conceptualisation of disability. Int. J. Adolesc. Youth.

[5] Kingdom of Saudi Arabia. General Authority of Statistics (2019). Population in the Ha’il Region by Gender, Age Group, and Nationality (Saudi/Non-Saudi). https://www.stats.gov.sa/en/6138.

[6] King Salman Center for Disability Research (2002). Disability Code Article 1.

[7] The Supreme Council (2002). The Supreme Council for the Affairs of Persons with Disabilities.

[8] Getzel E.E. (2008). Addressing the Persistence and Retention of Students with Disabilities in Higher Education: Incorporating Key Strategies and Supports on Campus. Exceptionality.

[9] Yousef R. (2019). Disability, Social Work and Social Exclusion: New Strategies for Achieving Social Inclusion of People with Physical Disabilities in the Kingdom of Saudi Arabia.

[10] Heaney C.A., Israel B.A. (2008). Social networks and social support. Health Behav. Health Educ. Theory Res. Pract..

[11] Leach J. (2014). Improving Mental Health through Social Support: Building Positive and Empowering Relationships.

[12] King G., Willoughby C., Specht J.A., Brown E. (2006). Social Support Processes and the Adaptation of Individuals With Chronic Disabilities. Qual. Health Res..

[13] Abuhamdah S.M.A., Naser A.Y., Abdelwahab G.M., AlQatawneh A. (2021). The Prevalence of Mental Distress and Social Support among University Students in Jordan: A Cross-Sectional Study. Int. J. Environ. Res. Public Health.

[14] Hassan S.-U., Algahtani F.D., Atteya M.R., Almishaal A.A., Ahmed A.A., Obeidat S.T., Kamel R.M., Mohamed R.F. (2021). The Impact of Extended E-Learning on Emotional Well-Being of Students during the COVID-19 Pandemic in Saudi Arabia. Children.

[15] Simadi F.A., Alqaryouti I.A. (2017). Students with disabilities’ satisfaction with their universities’ services. Int. J. Hum. Rights Healthc..

[16] Forouzan A.S., Mahmoodi A., Shushtari Z.J., Salimi Y., Sajjadi H., Mahmoodi Z. (2013). Perceived Social Support Among People With Physical Disability. Iran. Red Crescent Med. J..

[17] Onalu C., Nwafor N., Morese R., Auriemma V., Palermo S. (2022). Social Supports Available to Persons with Disabilities in Nigeria. Evolutionary Psychology Meets Social Neuroscience.

[18] Bromley K.W., Murray C., Rochelle J., Lombardi A. (2020). Social Support Among College Students With Disabilities: Structural Patterns and Satisfaction. J. Stud. Aff. Res. Pract..

[19] Lombardi A., Murray C., Kowitt J. (2016). Social support and academic success for college students with disabilities: Do relationship types matter?. J. Vocat. Rehabil..

[20] Hunt X., Bradshaw M., Vogel S.L., Encalada A.V., Eksteen S., Schneider M., Chunga K., Swartz L. (2022). Community Support for Persons with Disabilities in Low- and Middle-Income Countries: A Scoping Review. Int. J. Environ. Res. Public Health.

[21] Meleo-Erwin Z., Kollia B., Fera J., Jahren A., Basch C. (2020). Online support information for students with disabilities in colleges and universities during the COVID-19 pandemic. Disabil. Health J..

[22] Denisova O., Lekhanova O., Gudina T. (2020). Problems of distance learning for students with disabilities in a pandemic. SHS Web Conf..

[23] Estrada M., Zhi Q., Nwankwo E.M., Gershon R. (2019). The Influence of Social Supports on Graduate Student Persistence in Biomedical Fields. CBE—Life Sci. Educ..

[24] Williams P., Barclay L., Schmied V. (2004). Defining social support in context: A necessary step in improving research, intervention, and practice. Qual. Health Res..

[25] Hamdan-Mansour A.M., Dawani H.A. (2007). Social Support and Stress Among University Students in Jordan. Int. J. Ment. Health Addict..

[26] Cutrona C.E., Suhr J.A. (1992). Controllability of stressful events and satisfaction with spouse support behaviours. Communic. Res..

[27] Dafaalla M., Farah A., Bashir S., Khalil A., Abdulhamid R., Mokhtar M., Mahadi M., Omer Z., Suliman A., Elkhalifa M. (2016). Validity and reliability of Arabic MOS social support survey. Springerplus.

[28] Hombrados-Mendieta M.I., Gomez-Jacinto L., Dominguez-Fuentes J.M., Garcia-Leiva P., Castro-Travé M. (2012). Types of social support provided by parents, teachers, and classmates during adolescence. J. Community Psychol..

[29] Ko H.-C., Wang L.-L., Xu Y.-T. (2013). Understanding the Different Types of Social Support Offered by Audience to A-List Diary-Like and Informative Bloggers. Cyberpsychology Behav. Soc. Netw..

[30] Cacciatore M., Raggi A., Pilotto A., Cristillo V., Guastafierro E., Toppo C., Magnani F.G., Sattin D., Mariniello A., Silvaggi F. (2022). Neurological and Mental Health Symptoms Associated with Post-COVID-19 Disability in a Sample of Patients Discharged from a COVID-19 Ward: A Secondary Analysis. Int. J. Environ. Res. Public Health.

[31] Ma M., Fang J., Li C., Bao J., Zhang Y., Chen N., Guo J., He L. (2020). The status and high risk factors of severe psychological distress in migraine patients during nCOV-2019 outbreak in Southwest China: A cross-sectional study. J. Headache Pain.

[32] Herbert J.T., Hong B.S.S., Byun S., Welsh W., Kurz C.A., Atkinson H.A. (2014). Persistence and graduation of college students seeking disability support services. J. Rehabil..

[33] Al-Hendawi M., Thoma C.A., Habeeb H., Khair M.S. (2022). Emerging Themes on Factors Influencing Career and Employment Decisions: Voices of Individuals with Disabilities from Four Gulf Countries. Soc. Sci..

[34] Kim M.M., Kutscher E.L. (2020). College Students with Disabilities: Factors Influencing Growth in Academic Ability and Confidence. Res. High. Educ..

[35] Jesus T., Bhattacharjya S., Papadimitriou C., Bogdanova Y., Bentley J., Arango-Lasprilla J., Kamalakannan S. (2021). The Refugee Empowerment Task Force, International Networking Group of the American Congress of Rehabilitation Medicine Lockdown-Related Disparities Experienced by People with Disabilities during the First Wave of the COVID-19 Pandemic: Scoping Review with Thematic Analysis. Int. J. Environ. Res. Public Health.

[36] Dong S., Lucas M.S. (2013). An Analysis of Disability, Academic Performance, and Seeking Support in One University Setting. Career Dev. Transit. Except. Individ..

[37] Zavatkay D. (2015). Social Support and Community College Student Academic Persistence.

[38] Salgado S., González-Suhr C., Nazar G., Alcover C.-M., Ramírez-Vielma R., Bustos C. (2022). Relationships between Individual and Social Resources, Anxiety and Depression in the Early Lockdown Stage by the COVID-19 in Chile. Behav. Sci..

[39] Easop B.A. (2022). Education equity during COVID-19: Analyzing in-person priority policies for students with disabilities. Stan. L. Rev..

[40] Alqarni T., Algethami R., Alsolmi A., Adhabi E. (2019). College Students’ Knowledge and Attitudes toward the Inclusion of Persons with Disabilities in the University. Education.

[41] Gautam M., Thakrar A., Akinyemi E., Mahr G. (2020). Current and Future Challenges in the Delivery of Mental Healthcare during COVID-19. SN Compr. Clin. Med..

[42] Alsalem M.A. (2021). Towards New Disability Paradigms: Generating Equality in Saudi Arabian Policy in Light of the Convention on the Rights of Persons with Disabilities. Int. J. Disabil. Dev. Educ..

