# Supplementary material for: Types and Sources of Social Support Accessible to University Students with Disabilities in Saudi Arabia during the COVID-19 Pandemic
Source: Healthcare (Basel). 2023 Feb 6;11(4):464. doi: 10.3390/healthcare11040464 (PMC9957490; doi:10.3390/healthcare11040464)
Supplement: Supplementary file 1 [file healthcare-11-00464-s001.zip › healthcare-2181203-Supplementary.pdf]

Supplementary File A

Table S1. Alpha Reliability on Types of Social Support Scale

Social Support Sub-Scales*	Sample Items	Alpha Reliability
Information Support	My friends guide me toward all projects.	0.74
Emotional Support	I feel cared for and accepted by those around me.	0.71
Esteem Support	My colleagues appreciate my knowledge.	0.73
Social Integration Support	My friends share my interests in university life.	0.77
Tangible Support	My mother and family help me to take care of my needs.	0.72

*Each sub-scale comprises of five items rated on a three-point scale

Table S2. Sources of Social Support

Sources of Social Support Subscale*	Sample Items	Alpha Reliability
Support from Family	My family encourages me to continue my studies.	0.74
Support from Friends	My friends share my joys and sorrow.	0.72
Support from Teachers	My teacher encourages me to continue to share my needs.	0.71
Support from Colleagues	My colleagues appreciate my work.	0.71

*Each sub-scale comprises of five items rated on a three-point scale

Normality Assumption Check
Types of Social Support

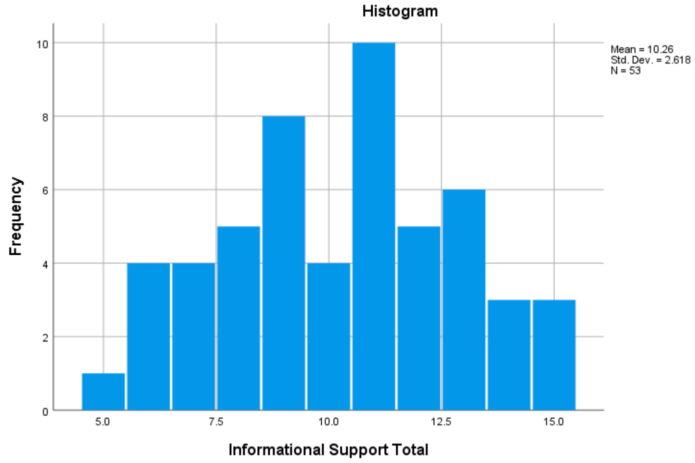

Figure S1. Informational Support

Table S3. Tests of Normality for Informational Support

	Kolmogorov-Smirnov[a]			Shapiro-Wilk		
**Tests of Normality**	Statistic	df	Sig.	Statistic	df	Sig.
Informational Support	.120	53	.061	.968	53	.159

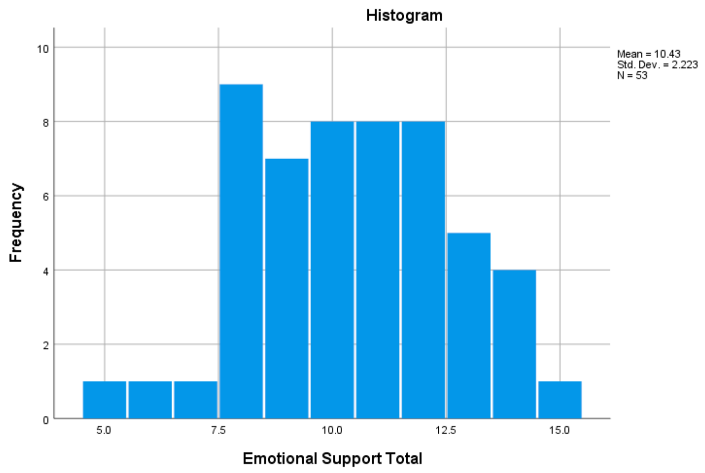

Figure S2. Emotional Support

Table S4. Tests of Normality for Emotional Support

Tests of Normality	Kolmogorov-Smirnov[a]			Shapiro-Wilk		
	Statistic	df	Sig.	Statistic	df	Sig.
Emotional Support Total	.099	53	.200*	.972	53	.240

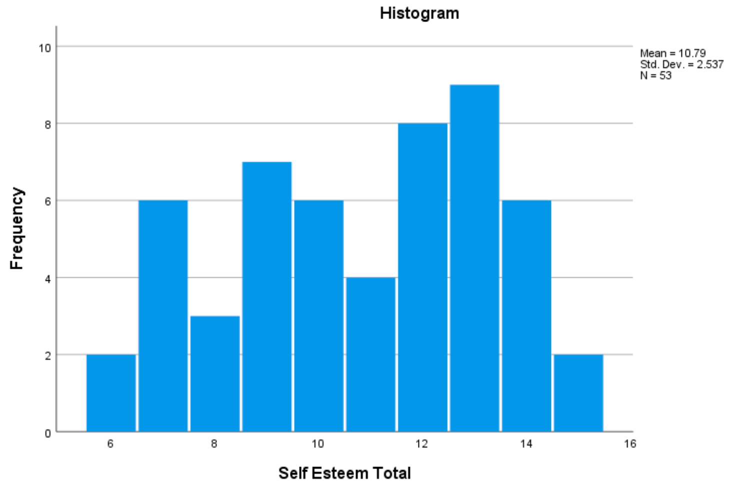

Figure S3. Self-Esteem Support (without transformation)

Table S5. Tests of Normality for Self-Esteem Support

Tests of Normality	Kolmogorov-Smirnov[a]			Shapiro-Wilk		
	Statistic	df	Sig.	Statistic	df	Sig.
Self Esteem Total	.155	53	.003	.943	53	.013

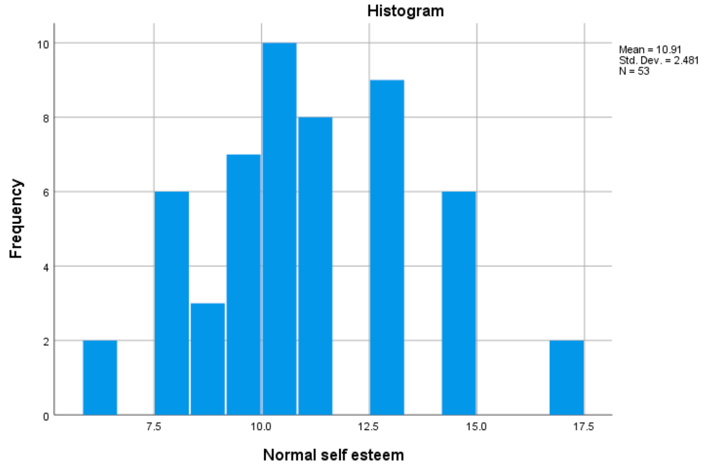

Figure S4. Self-esteem Support after transformation

Table S6. Tests of Normality for Self-Esteem Support after transformation

	Kolmogorov-Smirnov[a]			Shapiro-Wilk		
**Tests of Normality**	Statistic	df	Sig.	Statistic	df	Sig.
Normal self esteem	.085	53	.200*	.972	53	.253

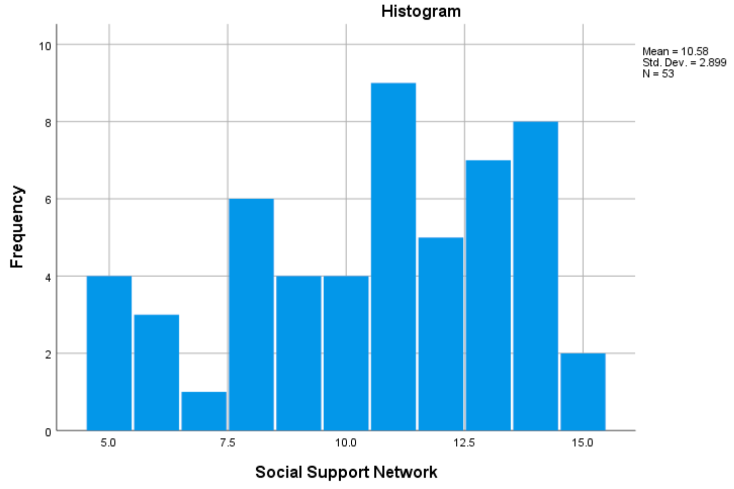

Figure S5. Social Integration Support (Without Transformation)

Table S7. Tests of Normality for Social Integration Support without transformation

	Kolmogorov-Smirnov[a]			Shapiro-Wilk		
**Tests of Normality**	Statistic	df	Sig.	Statistic	df	Sig.
Social Integration Support	.142	53	.010	.935	53	.006

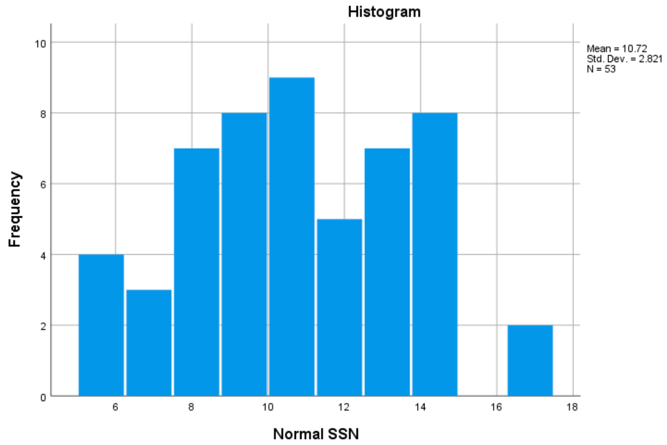

Figure S6. Social Integration Support (After Log transformations)

Table S8. Social Integration Support (After log transformation)

**Tests of Normality**	Kolmogorov-Smirnov[a]			Shapiro-Wilk		
	Statistic	df	Sig.	Statistic	df	Sig.
Normal SSN	.094	53	.200*	.967	53	.153

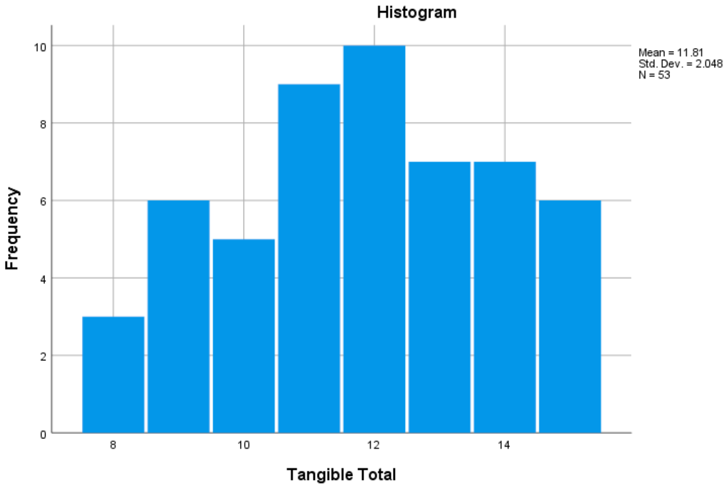

Figure S7. Tangible Support (Without log transformations)

Table S9. Tangible Support (Without log transformations)

**Tests of Normality**	Kolmogorov-Smirnov[a]			Shapiro-Wilk		
	Statistic	df	Sig.	Statistic	df	Sig.
Tangible Total	.103	53	.200*	.948	53	.022

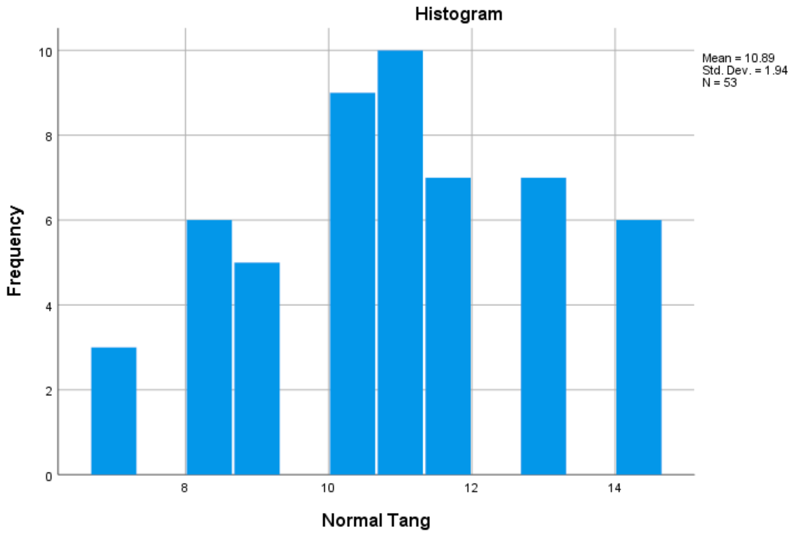

Figure S8. Tangible Support (After transformation)

Table S10. Tangible Support (After transformation)

**Tests of Normality**	Kolmogorov-Smirnov[a]			Shapiro-Wilk		
	Statistic	df	Sig.	Statistic	df	Sig.
Normal Tang	.099	53	.200*	.957	53	.064

Normality Assumption Check
Sources of Social Support

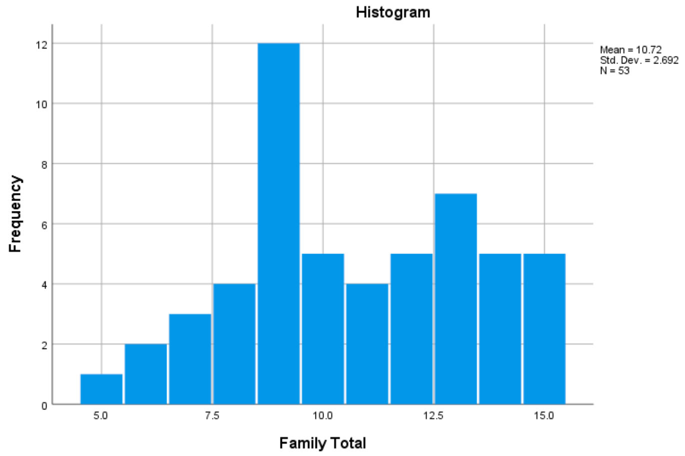

Figure S9. Support from Family

Table S11. Support from Family

**Tests of Normality**	Kolmogorov-Smirnov[a]			Shapiro-Wilk		
	Statistic	df	Sig.	Statistic	df	Sig.
Family Total	.153	53	.003	.951	53	.029

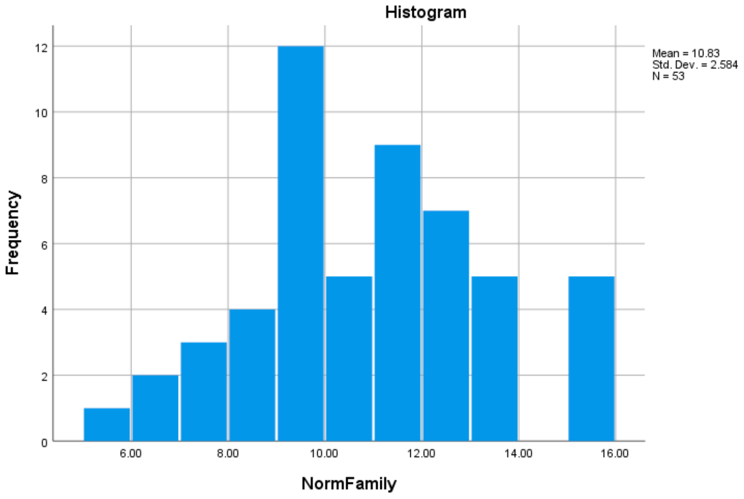

Figure S10. Family Support (After log transformation)

Table S12. Family Support (After log transformation)

Tests of Normality	Kolmogorov-Smirnov[a]			Shapiro-Wilk		
	Statistic	df	Sig.	Statistic	df	Sig.
Norm Family	.125	53	.038	.968	53	.172

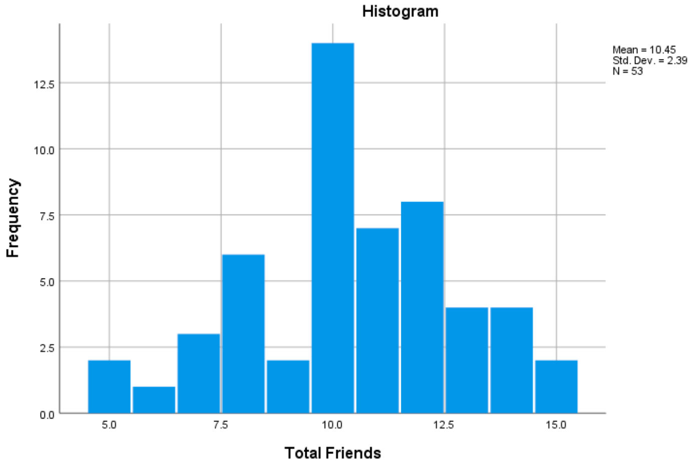

Figure S11. Support from friends

Table S13. Support from friends

Tests of Normality	Kolmogorov-Smirnov[a]			Shapiro-Wilk		
	Statistic	df	Sig.	Statistic	df	Sig.
Total Friends	.161	53	.002	.967	53	.147

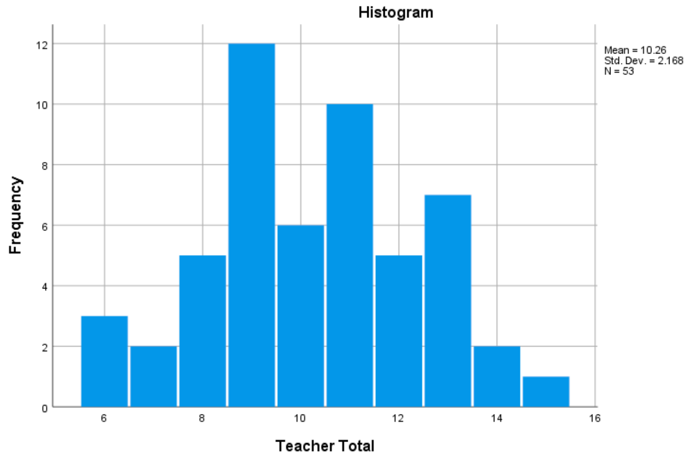

Figure S12. Support from teachers

Table S14. Support from teachers

Tests of Normality	Kolmogorov-Smirnov[a]			Shapiro-Wilk		
	Statistic	df	Sig.	Statistic	df	Sig.
Teacher Total	.135	53	.017	.967	53	.156

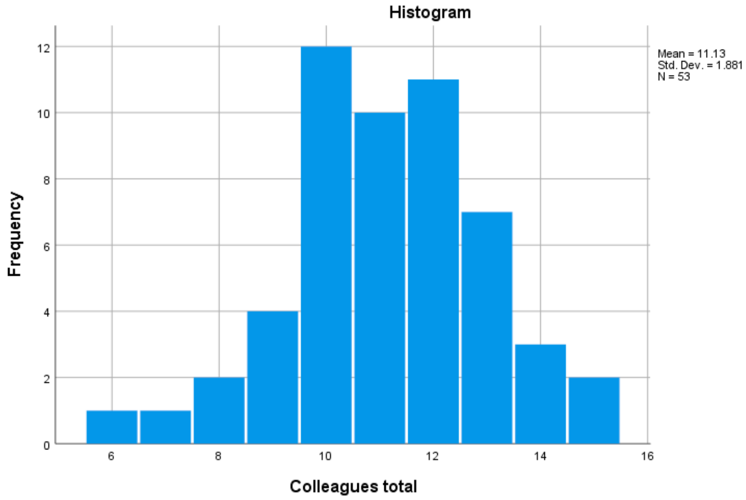

Figure S13. Support from colleagues

Table S15. Support from colleagues

Tests of Normality	Kolmogorov-Smirnov[a]			Shapiro-Wilk		
	Statistic	df	Sig.	Statistic	df	Sig.
Colleagues total	.123	53	.045	.968	53	.168
